# The Legislature and the Doctors

**Published:** 1895-04

**Authors:** 


### EDITORIAL DEPARTMENT.

F. E. DANIEL, M. D., Editor.
S. E. HUDSON, M. D., Managing Editor.
A. J. SMITH. M. D., Galveston, Associate Editor.

EDITORIAL STAFF:

PROF. J. E. THOMPSON, M. D., Texas Medical College, Galveston; Surgery.
PROF. WM. KEILLER, M. D., Texas Medical College, Galveston; Obstetrics and Gynecology.
PROF. DAVID CERNA, M. D., Texas Medical College, Galveston; Therapeutics.
PROF. A. J. SMITH, M. D., Texas Medical College, Galveston; Medicine
DR. R. H. L. BIBB, Saltillo, Mexico; Foreign Correspondent.

Official organ of the West Texas Medical Association, the Houston District Medical Association, the Austin District Medical Society, the Galveston County Medical Society, and several others.

### THE LEGISLATURE AND THE DOCTORS.

That there is a want of undertanding between the medical profession of the State and the legislators, has been manifest many times. The JOURNAL earnestly believes the repeated failure to secure medical legislation is to be attributed to this, rather than to any want of respect for the profession, or want of intellectual capacity on the part of the members, as has been charged. True, there is, in every legislature, a certain element who think it smart to ridicule the efforts of the profession, and in the present session this element is represented by a little pudgy fellow serving his first term, with head the size, shape and color of a cocoanut, and eyes like a 'possum's,—he who ridiculed Dr. Wooten's bill, and amended by exempting the old lady with her "yarb tea" (whereupon his sort went into paroxysms of hilarious hilarity and bubbled over with suppressed mirth); but the majority are sober, serious men, who, in our opinion, want to do right.

The profession, in our judgment, are themselves partly to blame for the repeated failure to secure medical legislation. In the first place, as the JOURNAL has before pointed out, the title of our bill has always belied our intentions. A bill to regulate the practice of medicine conveys to the mind the idea of control, and creates a suspicion that we want to monopolize the practice. A senator asked the writer if we proposed to regulate the practice of those of other schools; and when it was explained to him that the bill was an effort to protect the public from the ignorant and incompetent, he remarked, "That is not what your bill asks for."

Dr. Wooten's bill did not require an examination, and therein, had it passed, would have failed of the desired end. It provided for the acceptance of a *diploma* as *prima facie* evidence of qualification, and as *license to practice*,—but that diploma must have been granted by a college requiring not less than three years, of three sessions of not less than six months each, as a condition to graduation. In this there is an element of equity, in that as Texas requires so much of her own graduates, no State should have the right to foist its graduates upon us, turned loose with less preparation. Dr. Wooten did not ask for this bill because he believed that it filled the requirements, but because, in the light of past experience, he thought it was the best we could do; it is the only form of bill the homeopaths and eclectics will not oppose. They dread the very mention of examination, and fight any bill that provides for it.

It is perhaps best that it failed, because, while it would have been some improvement on the one we have, it would not entirely remedy the evil, and for our part, we insist on an efficient bill, or none, and we believe, in time, we will get it;—it will take time,—we must educate a legislature up to the requirements, undeceive them on many points, and make them understand many things they do not now understand. And to do this, we must begin at the beginning. Here is what we propose:

When candidates are announced for the next legislature, let the medical men pledge themselves to vote for no man who will not agree, after having been enlightened on the subject, to support a bill to *suppress the indiscriminate practice of medicine*, a bill restricting the privilege of practicing medicine to those who can show to a legally organized authority that they are qualified to practice, no matter how, when or where they acquired the knowledge, nor *how long it took them*. Let the aspirant for legislative honors be disabused of the prevalent idea that a diploma is *a license*. Let him know that a diploma is only "evidence of a de-degree having been conferred"—only presumptive evidence of qualification, it matters not whether from a two or a four years school, and, according to repeated court decisions, *conveys no rights whatever;* that to practice medicine is a *privilege*,—one which the State, in the exercise of her police powers for the protection of the public health, has a right to grant or refuse; and that it is a privilege fraught with such dangers and responsibilities that it should have every safeguard thrown around it.

It would seem a self-evident proposition that a man, ignorant of the human system and its economy, and ignorant of the properties of drugs, should not be permitted either to prescribe or dispense drugs that may, and often do, destroy life.

Disabuse the candidate's mind of the belief that seems to have possessed every legislative body that has assembled at Austin for ten years at least,—that a selfish motive underlies our efforts. Make them see that a broad humanitarian principle,—a desire for the well being and the protection of our fellow man, and not a selfish motive, actuates the profession in thus pointing out the danger, and asking the legislature for protection; and that the reason the people themselves do not ask for protection, and the profession do so for them, is that the people are in blissful ignorance of this great danger; the oily-tongued sharper may kill with impunity, and deceive the bereaved parent or husband or wife. They do not appreciate the danger, nor know the need of protection, any more than the orphan knows the need of the guardian appointed by the court.

Were this done, and pledges of support of some such bill as will remedy this crying evil of quackery and degradation of the very name of medicine, required as a condition for the support of the medical men at the polls, our next legislature would be composed of men ripe for and pledged to grant the request, and we may confidently hope for success, despite a sprinkling of Simpsons, who may never be enlightened.

